# Involvement of the TRPML Mucolipin Channels in Viral Infections and Anti-viral Innate Immune Responses

**DOI:** 10.3389/fimmu.2020.00739

**Published:** 2020-04-29

**Authors:** Giorgio Santoni, Maria Beatrice Morelli, Consuelo Amantini, Massimo Nabissi, Matteo Santoni, Angela Santoni

**Affiliations:** ^1^Immunopathology Laboratory, School of Pharmacy, University of Camerino, Camerino, Italy; ^2^Immunopathology Laboratory, School of Biosciences and Veterinary Medicine, University of Camerino, Camerino, Italy; ^3^Medical Oncology Unit, Hospital of Macerata, Macerata, Italy; ^4^Department of Molecular Medicine, Sapienza University, Rome, Italy; ^5^IRCCS Neuromed, Pozzilli, Italy

**Keywords:** TRP channel, mucolipin, innate immunity, viral infection, endolysosome

## Abstract

The TRPML channels (TRPML1, TRPML2, and TRPML3), belonging to the mucolipin TRP subfamily, primary localize to a population of membrane-bonded vesicles along the endocytosis, and exocytosis pathways. Human viruses enter host cells by plasma membrane penetration or by receptor-mediated endocytosis. TRPML2 enhances the infectivity of a number of enveloped viruses by promoting virus vesicular trafficking and escape from endosomal compartment. TRPML2 expression is stimulated by interferon and by several toll like receptor (TLR) activators, suggesting a possible role in the activation of the innate immune response. Noteworthy, TRPML1 plays a major role in single strand RNA/DNA trafficking into lysosomes and the lack of TRPML1 impairs the TLR-7 and TLR-9 ligand transportation to lysosomes resulting in decreased dendritic cell maturation/activation and migration to the lymph nodes. TRPML channels are also expressed by natural killer (NK) cells, a subset of innate lymphocytes with an essential role during viral infections; recent findings have indicated a role of TRPML1-mediated modulation of secretory lysosomes in NK cells education. Moreover, as also NK cells express TLR recognizing viral pattern, an increased TLR-mediated activation of cytokine production can be envisaged, suggesting a dual role in the NK cell-mediated antiviral responses. Overall, TRPML channels might play a double-edged sword in resistance to viral infections: on one side they can promote virus cellular entry and infectivity; on the other side, by regulating TLR responses in the various immune cells, they contribute to enhance antiviral innate and possibly adaptive immune responses.

## Discovery and Characterization of Mucolipin Channels

The transient receptor potential mucolipin channels (TRPML) are non-selective cation channels that conduct Ca^2+^ and monovalent cation currents from the lumen to the cytoplasm (1, 2). These channels are tetramers, consisting of proteins with six transmembrane-spanning domains and amino- and carboxy-terminal tails oriented toward the cytosol (3). Each subunit has six transmembrane segments (S1–S6) and a pore-loop between S5 and S6, which forms a voltage-sensor-like domain and a pore domain. A long extracellular linker between S1 and S2, “polycystin-mucolipin domain” is identified (4). There are three TRPML subtypes sharing ∼40% amino acid sequence identity (2). TRPMLs play a role in membrane trafficking (1, 2, 5), autophagy (6, 7), exocytosis (8), and ion homeostasis (9).

The *MCOLN1* gene encoding TRPML1 is located on human chromosome 19. No splicing variants have been found in humans, whereas splice variants were described in mice (10). The *MCOLN2* gene encoding TRPML2 is located on human chromosome 1 and only one TRPML2 isoform showing 60% amino acid homology with TRPML1, has been detected in humans. The human *MCOLN3* gene maps on the short arm of chromosome 1.

TRPML1 is expressed in a number of tissues including adrenal gland, lung, bladder and placenta as well as in thymus, spleen and immune cells (11–13). Mutations in *MCOLN1* gene cause a lysosomal storage disorder called mucolipidosis type IV (MLIV). Over 95% of patients with MLIV have loss of functional mutations in MCOLN1 (11–13). Many patients carry mutations that introduce premature stop signals in *MCOLN1*, thus the TRPML1 protein is completely absent, or abnormally short and it lacks the ion conducting pore (13–15). Some patients show single point mutations in *MCONL1* that maintain the open reading frame but lead to a incorrect location or to the production of a TRPML1 inactive form (11–14, 16–18). TRPML2 mRNA is mainly detected in lymphocytes and other cells of the immune system (19). In addition, TRPML2 was found to be overexpressed in aggressive human glioblastoma (20). TRPML3 is mainly expressed in cochlear and vestibular sensory hair cells and melanocytes (21). Two TRPML3 spontaneous gain-of-function mutations (A419P and I362T) called varitint-waddler mutations cause deafness and coat color dilution in mice (22–26).

TRPML1 is activated by phosphatidylinositol-3,5-biphosphate (PtdIns(3, 5)P2) (15, 21, 27–29). Moreover, TRPML1 has an intraluminal loop whose protonation stimulates channel activation (24, 30, 31). It is inhibited by phosphatidylinositol-4,5-biphosphate (PtdIns(4, 5)P2), sphingomyelins, and lysosomal adenosine (28, 29). PtdIns(3, 5)P2 is able to activate also TRPML2 and TRPML3. Na^+^ removal or less acidic/neutral pH activate TRPML3 and TRPML2, respectively (32, 33). Among synthetic activators currently available ML-SA1 activates TRPML1, TRPML2, and TRPML3 in human; ML2-SA1 is TRPML2 specific; MK6-83 activates TRPML1 and TRPML3 (15, 21, 28, 32). There are several synthetic inhibitors (ML-SIs); however, they are unable to discriminate the TRPML isoforms from each other (7, 8). Therefore, PtdIns(3, 5)P2 seems to have a central role in activating the TRPML family. This is a low-abundance endolysosome-specific phosphoinositide, produced by PtdIns (3) P5-kinase (PIKfyve). In the immune response, PtdIns(3, 5)P2 is responsible for the fusion of phagosomes with lysosomes to form phagolysosomes, which are essential for the digestion of engulfed pathogens (11, 12, 21). It should be noted that the phagosome acidification is allowed because PtdIns(3, 5)P2 activates TRPML1 channel by directly binding to its N-terminus (34).

## Subcellular Localization of TRPML Channels

TRPMLs primarily localize to vesicles along the endocytosis and exocytosis pathways. TRPML1 is localized in the lysosome-associated membrane protein (Lamp-1)^+^ or Rab7^+^ late endosomal and lysosomal (LEL) compartment (2, 26, 35, 36). Late endosomes have an acidic pH of 5.5–6.0, and lysosomes have a more acidic pH of 4.5–5.0 (37–39), a necessary condition to maintain the activity of lysosomal hydrolases (40). The lysosomal localization of TRPML1 protein is likely mediated by clathrin adaptor AP2-dependent internalization from the plasma membrane and/or AP1/AP3-dependent trafficking from the trans-Golgi network (41). Moreover, TRPML1 functions as a key lysosomal Ca^2+^ channel controlling both lysosome biogenesis and reformation, crucial events for cellular homeostasis (42). TRPML1 also regulates focal exocytosis and phagosome biogenesis. Phagocytic ingestion of large particles activates a PtdIns(3, 5)P2- and Ca^2+^-dependent exocytosis pathway necessary for pseudopod extension and for leading to clearance of senescent and apoptotic cells *in vivo* (8).

Similar to TRPML1, TRPML2, and TRPML3 co-localize with Lamp-1 and Rab7 in the LEL compartment (41).

Antigen presentation is central in activating adaptive immunity and is mainly mediated by professional antigen-presenting cells including dendritic cells (DCs) and macrophages. In mouse macrophages TRPML1 co-localizes with the MHC-II molecules (43), and by heteromeric interactions with TRPML2 (44) that also contributes to MHC-II/antigen complex formation. The TRPML2^+^ vesicles colocalize with CD63, Lamp-1 and Lamp-3, and Rab11; they induce accumulation of LysoTracker (3, 45), indicating that a fraction of TRPML2 is present in LEL. Numerous proteins, including MHC-I, CD59, interleukin-2 receptor, β_1_-integrins, and many glycosylphosphatidylinositol-anchored proteins (GPI-APs), travel along the Arf6-regulated pathway (46–48), and co-localize with TRPML2. In addition, Arf6 mutations induce sequestration of TRPML2, MHC-I, and GPI-APs into the same enlarged vacuolar organelles (49), suggesting that TRPML2 uses the Arf6 pathway to cycle between the plasma membrane and recycling endosomes. TRPML2 overexpression induces a strong activation of Arf6, while the inactive form of TRPML2 (D^463^D/KK) delays the recycling of internalized GPI-APs back to the plasma membrane (49). TRPML2 has been also suggested to participate in the regulation of the lysosomal compartment of B-lymphocytes (45).

Much less is known about the localization and function of TRPML3. TRPML3 is localized in the ELs, early endosomal (EEs), and plasma membrane compartments. Moreover, TRPML3 regulates endocytosis, membrane trafficking and autophagy (50).

## General Mechanisms for Virus Entry Into Host Cells

Viruses have developed different mechanisms and molecules to interact with proteins, lipids and sugar moieties expressed on the surface of host cells, which generally trigger virion uptake through the endosomal system (51, 52). Different endocytic cell routes for virus entry have been reported (53–58). Numerous host factors are involved in the viral uptake, including coat proteins (clathrin and caveolin), scission factors (dynamin 2), and regulatory and trafficking factors (Ras, Ras-related C3 botulinum toxin substrate 1, cell division control protein 42 homolog, and phosphatidylinositol 3-kinase,RabGTPases, etc.) (59). Endocytosis is a dynamic process and involves recycling, trafficking, maturation and fusion of endocytic vesicles (60). Viruses that are running the endocytic gauntlet, need to escape the endosome before being recycled back into the extracellular space (61–63), or degradation in the lysosome. Thus, enveloped viruses (e.g., *Filoviridae*, *Arenaviridae*, and *Orthomyxoviridae*) fuse the viral envelope with an endosome membrane, releasing their genomic content into the cytoplasm. Non-enveloped viruses (e.g., *Adenoviridae*, *Parvoviridae*, and *Picornaviridae*) use membrane-modifying proteins which can physically pierce the endosomal membrane to allow release of the genomic content into the cytoplasm and receptor switching to facilitate the viral endosomal escape (64). Another feature of viral endosomal penetration is the ability to co-opt membrane damage responses of the target cell, by recruiting host phospholipases (*Picornavirus*) or inducing lysosomal/autophagosomal exocytosis (*Adenovirus*) (65).

Among the receptors involved in virus uptake and anti-viral immune responses, a role for TRPMLs in virus infection as well as in the activation of innate immune responses has recently been suggested.

## TRPMLs Enhance Virus Infectivity by Increasing the Trafficking Efficiency of Endocytosed Viruses

Recently, it has been found that TRPML2 channel is one of the interferon (IFN)-stimulating genes (ISGs). However, as several ISGs, TRPML2 enhances the infectivity of the yellow fever virus, the *Zika virus*, *the influenza A virus* (IAV) and the equine arteritis virus, while no effect on the Venezuelan equine encephalitis virus, respiratory syncytial virus, or vesicular stomatitis virus has been reported (65–67). Human A549 lung adenocarcinoma cells, stably transfected with TRPML2, result in enhanced IAV infectivity and infectious virus production. Moreover, knockout of TRPML2 in A549 and U-2 OS osteosarcoma cells caused a reduction of viral infection. In addition, treatment of THP-1 monocytes with IFN-γ, poly (I:C) or LPS (68, 69) enhanced TRPML2 protein expression.

Specifically, TRPML2 promotes virus trafficking from early to late endosomes and causes an enhanced viral release into the cytosol and a consequent escape from endosomal compartments; thus, it promotes a productive infection. This process requires TRPML2 channel activity, but doesn’t involve the antiviral IFN signaling pathways, and broadly is applied to enveloped RNA viruses that are transported to late endosomes by infection. TRPML2 doesn’t modulate antiviral signaling in IFN-responsive A549 cells: indeed, no differences in MX1, interferon induced transmembrane protein 3, or Interferon alpha inducible protein 27as well as IFNB1 and ISG induction were found in TRPML2-expressing cells infected with either IAV or *Sindbis virus*. Both IAV and *Sindbis virus* infections were enhanced by TRPML2. Accordingly, no IAV infection has been evidenced in TRPML2-DD/KK dominant negative mutant stably-transfected A549 cells. Similarly, TRPML3 increased IAV infection in ectopically expressing cells (65).

Overall, these data suggest that TRPML-mediated increase of viral infection is not linked to impairment of IFN or ISG induction.

During virus life cycle, TRPML2 expression increases at the early, but not at late post-entry stages. No major effects on adhesion of IAV on A549 cell surface or virus particle endocytosed cells, have been observed, whereas TRPML2 affected virus vesicular trafficking by promoting the efficiency of IAV trafficking to late endosomes or by preventing virion degradation (65), with more IAV fused within the endosomes in ectopic TRPML2-expressing cells. This effect has been evidenced only with viruses requiring TRPML2-dependent transport to endocytic carrier vesicles/late endosomes.

In this regard, a rare genetic variant of human TRPML2, which induces a lysine/glutamine or arginine change at 370 aa in TM3 and TM4 domains of TRPML2 protein (MCOLN2-K370Q), failed to enhance TRPML2-mediated viral infection when ectopically expressed. Intriguingly, the frequency of this variant is rather low (about 3%), but increases (11%) in some African populations (65).

## TRPML2 Channel Triggers Anti-Viral Innate Immune Responses

Innate immune activation is based on the ability of the host to recognize pathogens through specific pathogen recognition receptors such as TLR, NOD-like receptors, lectin-like receptors and RIG-1 receptors. Engagement of these receptors activates the production of cytokines, chemokines, and interferons that by binding to their cognate receptors, signal through the JAK-STAT pathways and transcriptionally induce hundreds of ISGs (66, 67). Recent evidence indicates that TRPML2 is expressed at low levels in resting RAW 264.7 macrophages, but its expression is strongly induced upon TLR activation, with no effect on TRPML1 or TRPML3 (68). These data have been also confirmed in bone marrow and alveolar macrophages as well as in microglia from mice treated with a panel of TLR activators, including zymosan (TLR2 ligand), PolyI:C (TLR3 ligand), LPS (TLR4 ligand), R-848 (TLR7/8 ligand), and Imiquimod (TLR7 ligand). Endogenous TRPML2 co-localizes with perinuclear vesicles that also contain the transferrin receptor and likely correspond to recycling endosomes.

It is therefore interesting to consider the implications of TRPML2 up-regulation during *in vivo* viral infections, when IFN is produced and triggers a number of responses. In non-immune cells, basal or IFN-induced TRPML2 expression may lead to enhanced viral uptake thus promoting virus infection. However, in immune cells expressing higher levels of basal TRPML2 (68, 70, 71), TRPML2-mediated increased viral uptake also results in increased PAMP receptor engagement and activation, stronger immune response, and subsequently improved viral clearance. In this regard, apilimod, an inhibitor of PIKfyve by functioning as activator of the TRPML2 channels, blocks the entry and the infection of the Ebola virus and the Marburg virus in Huh 7 liver, in Vero E6 kidney cells and in human primary macrophages. Apilimod also blocked Ebola-glycoprotein-virus like particle (VLP) entry and VLP infection (72).

Infection of mouse bone marrow derived macrophages (BMDM) with the intracellular *Mycobacterium smegmatis* induces TRPML2 expression, suggesting that TRPML2 up-regulation, occurs not only in response to purified TLR ligands but also to live pathogens. In addition, TRPML2 knocked-out mice treated for 24 h with LPS showed reduced expression of chemokine (C-C motif) ligand (CCL) 3, CCL5, chemokine (C-X-C motif) ligand 2 and Intercellular Adhesion Molecule 1. Moreover, the amount of secreted CCL2 a chemokine released via the early/recycling endosomalwas significantly reduced in the supernatants from LPS-treated TRPML2^–/–^ BMDM. Similarly, ML2-SA1 a new TRPML2 agonist, stimulated CCL2 release by LPS-activated WT but not TRPML2^–/–^macrophages (73). ML2-SA1 treatment also promoted macrophage migration (32), and macrophage and neutrophil migration, in response to LPS, was reduced in TRPML2 knocked-out mice (68).

## Involvement of TRPML1 in the Regulation of TLR Responses in DC: Potential Role in the Antiviral Adaptive Immunity?

Dendritic cells play an important role in the beginning of specific immune responses. Immature DCs patrol the tissues and check for the antigen presence by continuously internalizing extracellular material mainly via micropinocytosis (32, 74). Sensing of pathogen/danger signals triggers the DC maturation, reduces the antigen uptake, and up-regulates the membrane expression of CC-chemokine receptor 7 that binds to CCL21 and CCL19 chemokines, driving DCs to lymph nodes, where they present the antigen to T cells (75, 76). Recent evidence indicates that activating the TRPML1-transcription factor EB (TFEB), by regulating TRPML1 gene expression, allows DCs to switch from a tissue-patrolling mode to a fast migratory mode in order to reach the lymph nodes (77). Upon microbial sensing, lysosomal calcium is released by TRPML1, which in turn activates myosin II at the cell rear, promoting fast and directional migration. Lysosomal calcium also induces the activation of TFEB, that at steady state is phosphorylated by the mammalian target of rapamycin complex 1 (mTORC1) and remains in the cytosol (78), but due to the dephoshorylation induced by the TRPML1-mediated calcium efflux-activated calcineurin (79), TFEB translocates into the nucleus where it regulates the TRPML1 expression. Of interest, sensing of bacterial or viral products also induced the TFEB translocation from the cytosol to the nucleus with consequent expression of a network of genes involved in lysosome activity, biogenesis, and secretion (80–83).

Toll like receptors play a crucial role in the early host detection of invading viruses (84–87). In particular, TLR-7 and TLR-9 recognize single-stranded RNA (ssRNA) and double-stranded RNA (dsRNA), respectively, in the endolysosomes (84–86). A recent report demonstrates the involvement of TRPML1 in TLR7-mediated DC responses by faciliting ssRNA trafficking into lysosomes. TRPML1^–/–^ DCs showed impaired TLR7 responses to ssRNA, while a mucolipin agonist specifically enhanced TLR7 responses to ssRNAs. In addition, the inhibition of PtdIns(3, 5)P2 generation, that binds directly to TRPML1 and induces the Ca^2+^ release, completely inhibited TLR7 responses to ssRNA in DCs (88). Confocal analyses showed that ssRNA transportation to lysosomes in DCs was impaired by a PIKfyve inhibitor as well as by the lack of TRPML1. Moreover, in TRPML1^–/–^bone marrow derived-DCs (BM-DCs) RNA transportation to lysosomes was more severely impaired than DNA transportation. TLR9 responses to CpG-A were also significantly impaired in TRPML1^–/–^ Bone Marrow-conventional DCs (BM-cDCs) and plasmocytoid-DC (pDCs) by the PIKfyve inhibitor, suggesting that TRPML1 has a role in CpG-A transportation to lysosomes. However, CpG-A transportation to lysosomes was only transiently halted in TRPML1^–/–^ BM-cDCs, suggesting a redundant role of TRPML1 in this pathway. Conversely, TLR9 responses to CpG-B were not altered in TRPML1^–/–^ BM-cDCs and pDCs (77, 88). Impaired TLR7 and TLR9 responses in TRPML1^–/–^ BM-cDCs stimulated with ssRNAor CpG-Awas associated with reduced IL-6, TNF-α and IFN-α production. In addition, the PIKfyve inhibitor induced an impairment of TLR7 responses to ssRNA in BM-pDCs, while only reduced production of IFN-α in response to TLR9 stimulation by CpG-A was observed.

A role for TRPML channels in CpG-A transportation is not limited to TRPML1, but it has been described also for TRPML2 and TRPML3 (88).

Collectively, these findings suggest that TRPML channels, by enhancing TLR responses and promoting DC maturation/activation, play a critical role in stimulating anti-viral adaptive immune responses. In this regard, it has recently been discovered that TRPML2 increases the expression of B7 costimulatory molecules on DC via TFEB activation and simultaneously induces CD8 T cell proliferation and cytolytic activity in an antigen-specific manner (89).

## TRPML1 and TRPML2 Regulate of NK Cell Functions: a Dual Role in Antiviral NK Cell Responses?

Natural killers are a subset of innate lymphoid cells that have the ability to recognize and eliminate infected cells. Moreover, they can secrete anti-viral cytokines such as IFN-γ and TNF-α and chemokines to recruit and instruct other immune cell types (87). The activation of NK cell depends on a delicate balance between activating and inhibitory signals, the latter mainly being transduced by (KIRs, CD94/NKG2A) receptors for class I MHC. The interaction of MHC I inhibitory receptors with their self-ligands also results in the acquisition of the effector potential, a process called NK cell education (90, 91). The recognition of abnormal self on virus-infected cells triggers a number of non-MHC I-restricted activating receptors such as NKG2D, MHC I-related molecules MHC class I chain-related protein A and B (MICA and MICB) and UL16 binding proteins (ULBPs), DNAX Accessory Molecule-1and the NCR. These activating receptors function mainly in a cooperative manner to overcome the inhibitory signals of KIR and CD94-NKG2A receptors (92).

As other innate immune effector cells, NK cells can also sense viral patterns by means of TLRs. Indeed TLR3, TLR 7, TLR 8, and TLR 9 have been detected in human NK cells and the engagement by their respective ligands leads to production IFN-γ and CSFs and chemokines. Moreover, antiviral NK cell-mediated reactivity strongly relies on the cross-talk with other innate immune cells, including DCs and macrophages which can promote NK cell effector functions and proliferation by secreting cytokines such as IFN-α/β, IL-12, and IL-15, respectively (93–95).

Recent findings demonstrated that TRPML1 is expressed at mRNA level in both educated (KIR^+^) and not educated (KIR^–^) NK cell subsets. Interestingly, pharmacological inhibition of PtdIns(3, 5)P2 synthesis, or genetic silencing of TRPML1, resulted in enlargement of lysosomal compartment, increased granzyme B (GZB), and enhanced specific degranulation and IFN-γ production. On the contrary, stimulation of NK cells with the TRPML agonist, MK6-83, induced the loss of GZB and decreased degranulation and IFN-γ production in response to K562 cells. Overall, these findings suggest an important role of TRPML1-mediated modulation of secretory lysosomes in NK cell education (96). Recent evidence reported that NK cells also express high levels of TRPML2, which further increase during NK cell differentiation. Silencing of TRPML2 leads to slight enhanced NK cell degranulation and to the production of IFN-γ (96).

Finally, although no evidence is available in the literature so far, a role for TRPML in promoting NK cell-mediated TLR responses to viral patterns can be envisaged (97).

Collectively, these findings suggest that TRPML channels negatively affecting NK cell education and promoting TLR activation, play a dual role in NK cell-mediated antiviral responses.

## Conclusion

**FIGURE 1:**
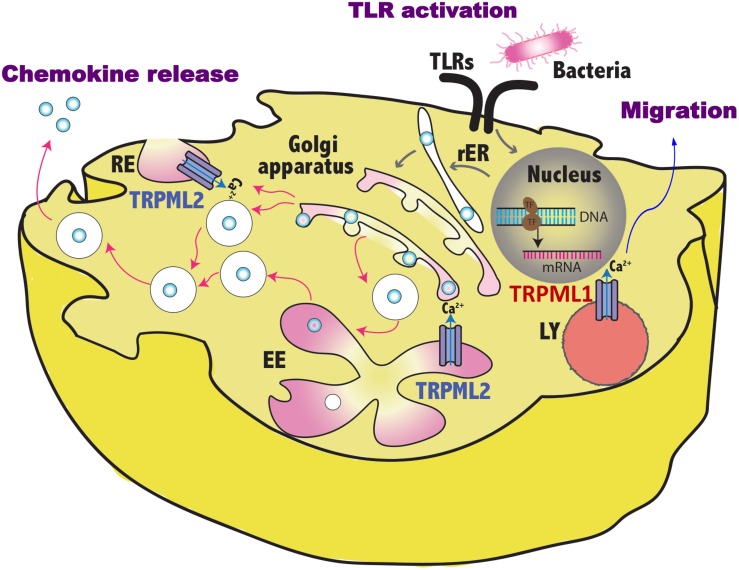
Schematic representation of TRPML1 and TRPML2 involvement in the regulation of vesicles trafficking induced by pathogen sensing. By controlling the fluxes of vesicular Ca^2+^, TRPML1 promotes in dendritic cells the activation of myosin II that leads to fast and directional migration whereas TRPML2 regulates in macrophages the fission/fusion processes of transport vesicles and so the release of chemokines in the environment. TLR, toll like receptor; RE, recycling endosome, LY, lysosome; EE, early endosome; rER, rough endoplasmic reticulum.

Several evidences indicate that TRPMLs play a crucial role in membrane trafficking, autophagy, exocytosis and ion homeostasis. Thanks to these functions, TRPML1 and TRPML2 have been found to be involved in the entry and trafficking of virus by promoting virus infectivity and productive infection. Conversely, these receptors are also expressed on innate immune cells where they stimulate the transport of viral patterns and therefore the cognition by their respective receptors present in the endosomal compartment. In DCs, this results in enhanced TLR responses that lead to increased DC maturation, production of IFNs, inflammatory cytokines/chemokines and migration with consequent activation of the anti-viral adaptive immune responses (Figure 1). Recent findings indicate that on NK cells, which also express TRPML1 and TRPLM2 as well as the TLRs recognizing the viral nucleic acids, TRPML1 impairs NK cell education and functional activity by modulating the secretory lysosomes, thus suggesting a dual role in the NK cell-mediated antiviral responses.

For what concern TRPML3 role in infections, it has been demonstrated in bladder epithelial cells that TRPML3, by mediating efflux of Ca^2+^ ions from lysosomes, promotes the expulsion of exosome-encased bacteria (98). However, little is known about its functions in viral infections. At this regard, findings showed that TRPML3 increased IAV infections in ectopically expressing cells (65). Moreover, it has been recruited in the autophagosome upon induction of autophagy (50) and this suggests that it could participate to the xenophagy. Regarding the viral infections, autophagy can be either pro-viral or anti-viral. Some virus exploit the autophagy machinery for their intracellular survival, while other express specific protein to evade autophagy and propagate in host cells (99). Thus, an important role of TRPML3 in viral infections cannot be excluded; however, additional findings are required to further clarify this issue.

## Author Contributions

GS and AS drafted the manuscript. GS conceived and designed the study. MM, CA, MN, and MS critically revised the manuscript.

## Conflict of Interest

The authors declare that the research was conducted in the absence of any commercial or financial relationships that could be construed as a potential conflict of interest.
